# Protective Role of STAT3 in NMDA and Glutamate-Induced Neuronal Death: Negative Regulatory Effect of SOCS3

**DOI:** 10.1371/journal.pone.0050874

**Published:** 2012-11-30

**Authors:** Keun W. Park, Susan E. Nozell, Etty N. Benveniste

**Affiliations:** Department of Cell, Developmental, and Integrative Biology, University of Alabama at Birmingham, Birmingham, Alabama, United States of America; University of Louisville, United States of America

## Abstract

The present study investigates the involvement of the IL-6 family of cytokines, activation of the transcription factor Signal Transducer and Activator of Transcription-3 (STAT3), and the role of Suppressor Of Cytokine Signaling-3 (SOCS3) in regulating excitotoxic neuronal death *in vitro*. Biochemical evidence demonstrates that in primary cortical neurons and SH-SY5Y neuroblastoma cells, IL-6 cytokine family members, OSM and IL-6 plus the soluble IL-6R (IL-6/R), prevent NMDA and glutamate-induced neuronal toxicity. As well, OSM and IL-6/R induce tyrosine and serine phosphorylation of STAT3 in primary cortical neurons and SH-SY5Y cells. Studies using Pyridine 6 (P6), a pan-JAK inhibitor, demonstrate that the protective effect of OSM and IL-6/R on neuronal death is mediated by the JAK/STAT3 signaling pathway. In parallel to STAT3 phosphorylation, OSM and IL-6/R induce SOCS3 expression at the mRNA and protein level. P6 treatment inhibits SOCS3 expression, indicating that STAT3 is required for OSM and IL-6/R-induced SOCS3 expression. Lentiviral delivery of SOCS3, an inhibitor of STAT3 signaling, into primary neurons and SH-SY5Y cells inhibits OSM and IL-6/R-induced phosphorylation of STAT3, and also reverses the protective effect of OSM and IL-6/R on NMDA and glutamate-induced neurotoxicity in primary cortical neurons. In addition, treatment with IL-6 cytokines increases expression of the anti-apoptotic protein Bcl-xL and induces activation of the Akt signaling pathway, which are also negatively regulated by SOCS3 expression. Thus, IL-6/R and OSM-induced SOCS3 expression may be an important factor limiting the neuroprotective effects of activated STAT3 against NMDA and glutamate-induced neurotoxicity.

## Introduction

Members of the IL-6 cytokine family are key regulators of inflammatory and immunological responses, and include IL-6, IL-11, Cilliary Neurotrophic Factor (CNTF), Oncostatin M (OSM), Leukemia Inhibitory Factor (LIF), Cardiotrophin-1 (CT-1), and Cardiotrophin-Like Cytokine (CLC) [1]. The IL-6 cytokines are critical for fetal neurodevelopment, and participate in CNS neurodegenerative diseases [2]–[4]. IL-6 cytokine family signaling occurs when the cytokine binds to its cognate receptor, causing it to associate with gp130, the common signal transducing subunit for IL-6 cytokines. Formation of the ligand-receptor complex leads to activation of gp130-associated Janus Kinases (JAKs), which recruit and phosphorylate Signal Transducer and Activator of Transcription (STAT) proteins, predominantly STAT3. Phosphorylated STAT3 dimerizes and translocates to the nucleus where it induces transcription of target genes such as Bcl-xL, Bcl-2 and survivin, which are critical for promoting neuronal survival [5]. Astrocytes and microglia are the major source of IL-6 production in the CNS (as well as other IL-6 members) [6]–[8]. Constitutive release of IL-6 family member from these cells is minimal. Instead, stimulation by a variety of factors (cytokines, PGE_2_, neurotransmitters) is needed for cytokine production [6]–[8]. Neurons have the capacity to bind these cytokines and initiate signaling via expression of gp130 and ligand-specific receptors [9]–[11]. Activation of the STAT3 signaling pathway in neurons has been shown to promote neurite outgrowth and protect against neuronal death [4], [9], [10], [12]. In addition, leptin-induced STAT3 activation inhibits glutamate-induced neuronal death *in vivo* and *in vitro* [5]. These findings indicate that IL-6 cytokines contribute to neuroprotection, likely through STAT3 activation.

Suppressors Of Cytokine Signaling (SOCS) proteins function in a negative feedback loop to terminate signaling through the JAK/STAT pathway [13], [14]. SOCS1, SOCS2 and SOCS3 have been shown to regulate neuronal growth and differentiation [15], [16]. We focus on SOCS3 in this study. SOCS3 binds phospho-tyrosine residue 759 within gp130, subsequently inhibiting signal transduction [17], [18]. More recently, SOCS3 was shown to bind directly to JAK1 and JAK2 and inhibit tyrosine kinase activity [19]. Expression of SOCS3 in neurons plays a negative role in regulation of cell survival and neurite outgrowth [4], [10], [20], [21]. These studies demonstrate that cytokine or nerve injury-induced SOCS3 expression negatively regulates phosphorylation of STAT3, consequently leading to inhibition of neuron protection and neurite outgrowth.

In the present study, we demonstrate that three IL-6 cytokines, IL-11, IL-6/R and OSM, partially inhibit NMDA or glutamate-induced neuronal death and that this is mediated by STAT3 activation. IL-6 cytokines also induce SOCS3 expression and this results in inhibition of STAT3 phosphorylation in neurons, and a loss of the neuroprotective effects of IL-6 cytokines. SOCS3 also inhibits Bcl-xL expression and the Akt signaling pathway. Collectively, these results suggest that IL-6 cytokine-induced SOCS3 expression contributes to neuronal death by inhibiting the protective effect of activated STAT3.

## Materials and Methods

### Reagents

Recombinant human IL-11, CNTF, IL-6, sIL-6R and OSM were purchased from R&D Systems (Minneapolis, MN). Antibodies (Abs) against phospho-STAT3 Tyr705, phospho-STAT3 Ser727, STAT3, phospho-Akt Ser473, Akt, Bcl-xL and Bax were purchased from Cell Signaling Technology (Beverly, MA). Abs against GAPDH and GFAP were from Abcam (Cambridge, MA), MAP-2 Ab from Sigma-Aldrich (St. Louis, MO), and SOCS3 Ab from Santa Cruz Biotechnology (Santa Cruz, CA). NMDA and glutamate were purchased from Sigma-Aldrich, and Pyridine-6 (P6), a pharmacological inhibitor of JAKs [22], was purchased from Calbiochem (La Jolla, CA). The plasmid pLVX-IRES-ZsGreen was purchased from Invitrogen (Grand Island, NY).

### Primary Cortical Neuron Cultures and SH-SY5Y Cells

Animal studies have been approved by the University of Alabama at Birmingham IACUC. Primary murine cortical neuron cultures were prepared from embryonic day 17 C57BL/6J mice. Mice were first sacrificed by CO_2_ inhalation and embryos collected and neocortices were mechanically triturated as described previously [23]. Dissociated cells were plated on Poly-D-Lysine coated 6-well and 24-well plates at a density of 1×10^6^ and 5×10^4^ cells/well, respectively. Plating media consisted of Neurobasal Medium (Gibco, Carlsbad, CA) supplemented with 2% B-27, 0.5 mM GlutaMax, and 1% penicillin-streptomycin. Every 3 days *in vitro* (DIV), the medium was replaced with fresh media until DIV 11, at which time the cells were used. Purity of the cultures was confirmed by MAP-2 staining for neurons (>95% positive), and GFAP staining for astrocytes (4–5% positive; Figure S1). The human neuroblastoma cell line SH-SY5Y was purchased from ATCC (Manassas, VA) [24], and was grown in DMEM/F12 with 10% fetal bovine serum, 2 mM L-Glutamine, and 1% penicillin-streptomycin. For SH-SY5Y cell differentiation, cells were grown in fresh medium containing 10 µM all-trans retinoic acid (RA) was added for 5 days.

### Lentiviral Vector Production and Infection

For lentiviral expression of SOCS3, the human SOCS3 open reading frame was cloned into the the lentiviral vector, pLVX-IRES-ZsGreen to generate pLVX-IRES-ZsGreen/SOCS3 (Lenti-SOCS3). To knockdown SOCS3 expression, a lentiviral plasmid encoding shRNA specific for SOCS3, pGipz/shSOCS3, was purchased from Open Biosystems (Lafayette, CO). Lentiviral particles were generated by calcium phosphate-mediated cotransfection of HEK-293T cells with empty pLVX-IRES-Green or pLVX-IRES-Green/SOCS3, empty pGipz or pGipz/shSOCS3, psPAX2 (Packaging plasmid), and pMD2G (Envelope plasmid). Virus was collected after 72 h, and titers up to 3–4×10^6^ infectious units/ml were obtained. SH-SY5Y cells were infected with virus, sorted by FACS analysis using green fluorescence protein (GFP) expression, and maintained. Primary neurons were infected with virus at DIV 7 at a multiplicity of infection (M.O.I) of 40 and used at DIV 11.

### Immunoblotting

Cultures treated with cytokines were lysed in buffer containing the following: 150 mM NaCl, 10 mM Na_2_HPO_4_ (pH 7.2), 0.5% sodium deoxycholate, 1% NP-40, and protease inhibitor mixture. Forty µg of total cell lysate was separated by electrophoresis on 8% SDS-polyacrylamide gels and blotted with antibodies, as described previously [25]. Immunoreactivity was assessed using Pierce ECL® or SuperSignal® West Dura substrate (Thermo Scientific, Rockford, IL). For quantitative analyses, the densities of bands on immunoblots were measured with ImageJ software.

### RNA Isolation and Quantitative Realtime-PCR

Cultures treated with cytokines were washed with RNase-free PBS, and total RNA was extracted using Trizol (Invitrogen, San Diego, CA). One µg of purified RNA was reverse transcribed as previously described [26]. Quantitative real-time PCR (qRT-PCR) to determine levels of SOCS3 mRNA was performed as previously described [26]. The data were analyzed using the comparative cycle threshold method to obtain quantitation values.

### Measurement of Neurotoxicity

Neuronal cell death was analyzed by measuring the level of 3-(4,5-dimethylthiazol-2-yl)-2,5-diphenyl tetrazolium bromide (MTT) reduction, and lactate dehydrogenase (LDH) released into the bathing medium. For the MTT reduction assay, MTT was added to each culture post-treatment to a final concentration of 600 µM, and the cells were incubated for 3 h at 37°C. After incubation, medium was removed and DMSO was added to each well. The absorbance was measured with a microplate reader at a test wavelength of 570 nm and a reference wavelength of 655 nm, as previously described [23]. LDH release was assessed with the LDH assay kit (Promega, WI) according to the manufacturer’s protocol with some modifications. Briefly, 50 µl of culture medium was collected and incubated with substrate mix including 0.2 mg of β-NADH at room temperature. The absorbance at a wavelength of 340 nm was measured immediately after 0.1 M sodium pyruvate was added to the mixture. The unit activity of LDH was defined as decrease of A_340_. The percentage of neuronal death was normalized to the mean LDH value released after a sham control (defined as 0%) or continuous exposure to 500 µM NMDA for 24 h (defined as 100%).

### Statistical Analysis

All experiments were performed utilizing three different preparations of primary cortical neurons or three different passages of SH-SY5Y cells, and repeated at least three times. All values are expressed as mean ± SEM. Statistical significance (*p*<0.05 for all analyses) was assessed by ANOVA using GraphPad Prism 5.01 (GraphPad, San Diego, CA), followed by Student–Newman–Keuls analyses.

**Figure 1:**
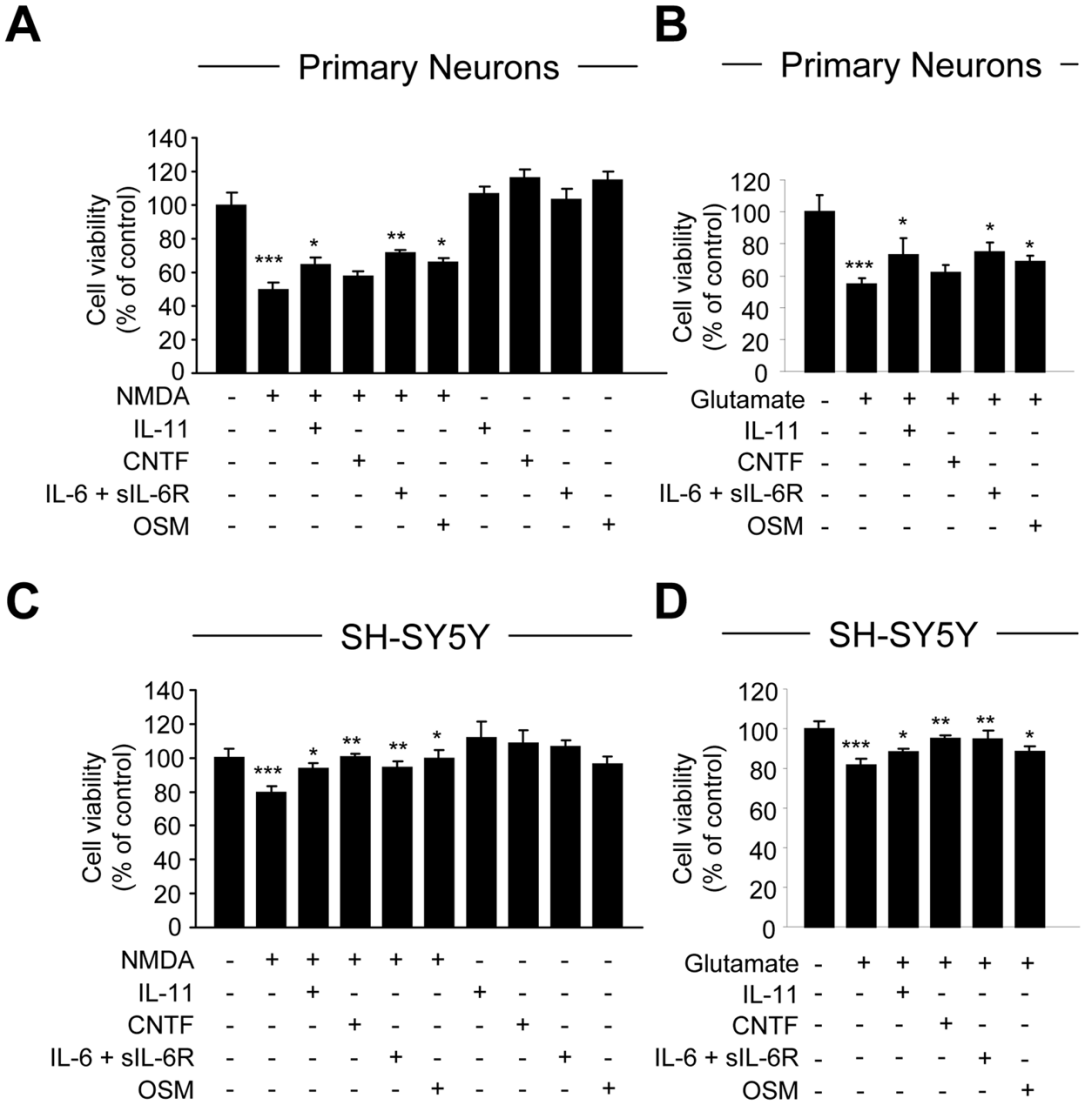
Neuroprotective Effect of IL-6 Cytokines on NMDA and Glutamate-induced Neuronal Death. Primary cortical neurons or SH-SY5Y cells were pretreated with IL-11 (10 ng/ml), CNTF (10 ng/ml), IL-6 (10 ng/ml) plus sIL-6R (25 ng/ml) or OSM (10 ng/ml) for 1 h, and then grown in the presence of cytokine and NMDA (100 µM, **A**) and (1 mM, **C**) or glutamate (100 µM, **B**) and (1 mM, **D**) for an additional 24 h. Cell viability was determined by the MTT reduction assay. Graphic representation of the mean ± SEM of triplicate cultures in three separate experiments. ***p<0.001 compared to control; *p<0.05 and **p<0.01 compared to NMDA or glutamate only.

## Results

### Neuroprotective Effect of IL-6 Cytokines on NMDA and Glutamate-induced Neuronal Death

IL-6 has been shown to inhibit glutamate release in the cerebral cortex [27] and suppress the neurotoxic effects of the glutamate analogue NMDA *in vitro* and *in vivo* [28], [29]. Recent reports have demonstrated that OSM, one member of the IL-6 cytokine family, protects against NMDA-induced cortical neuronal death *in vivo* and *in vitro* [30], [31]. Thus, we investigated whether treatment with other IL-6 cytokine family members, including IL-11, CNTF, IL-6 plus sIL-6R (IL-6/R) and OSM, protects against NMDA or glutamate-induced neuronal death. The IL-6R is found in both membrane-bound and soluble (sIL-6R) forms [32]. We previously demonstrated that the addition of sIL-6R with IL-6 induces optimal IL-6-mediated responses, compared to IL-6 alone [7], [26]. These IL-6 family members were chosen because they are elevated in numerous neurological disease states. Murine cortical neuron-enriched cultures were treated with 100 µM NMDA or 100 µM glutamate in the absence or presence of IL-11, CNTF, IL-6/R or OSM, and neuronal death was assessed using the MTT reduction assay. As shown in Figure 1A, a 24 h treatment of IL-11, CNTF, IL-6/R or OSM alone had no effect on neuronal viability, whereas a 24 h NMDA treatment induced ∼50% cell death. However, cells pretreated with IL-11, IL-6/R or OSM for 1 h, then continued cytokine exposure plus NMDA for 24 h, had a significant partial protective effect against NMDA-induced neuronal death by 14%, 16% and 22%, respectively (Figure 1A). Interestingly, CNTF did not exert a protective effect. In addition to NMDA, glutamate was tested to investigate whether the neuroprotective effect of IL-6 cytokines is excitotoxin specific. Glutamate-induced primary cortical neuronal death was partially inhibited by a 1 h pretreatment with IL-11, IL-6/R or OSM by 19%, 20% and 14%, respectively (Figure 1B). Again, CNTF did not protect against glutamate-induced neurotoxicity. As the cell numbers obtained from primary neuron cultures were limited, further studies to examine the underlying mechanism of cytokine-induced neuron protection against NMDA or glutamate toxicity were performed using the human neuroblastoma cell line SH-SY5Y. We observed similar results in differentiated SH-SY5Y cells. Treatment with NMDA (Figure 1C) or glutamate (Figure 1D) induced neuronal death by 20% and 21%, respectively. Pretreatment with IL-11, CNTF, IL-6/R or OSM protected against NMDA (Figure 1C) or glutamate (Figure 1D) induced death of SH-SY5Y cells. In contrast to primary neurons, CNTF was protective in SHSH-5Y cells.

**Figure 2:**
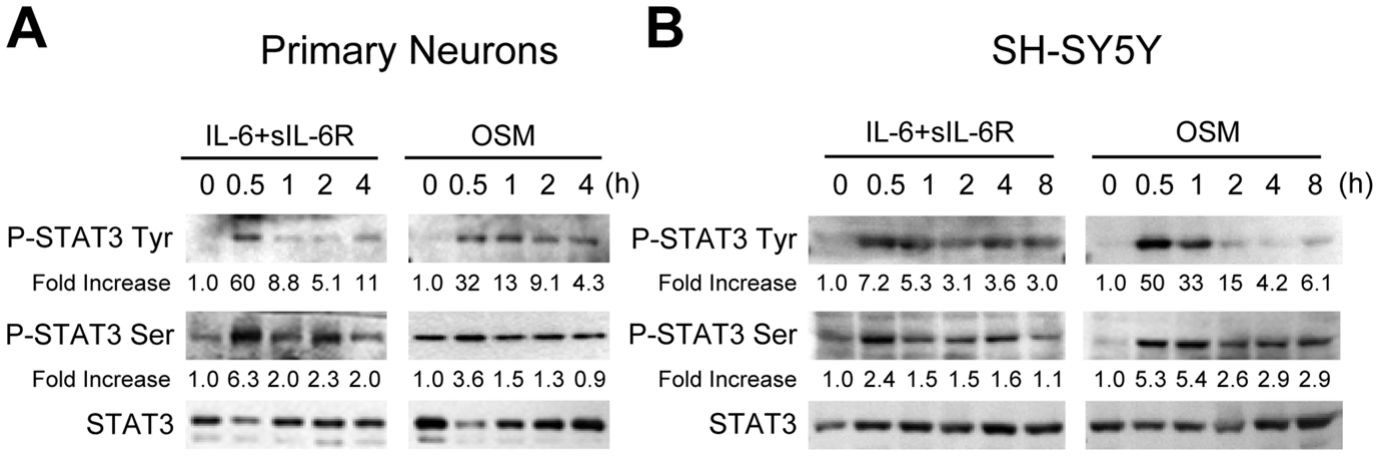
IL-6 Cytokines Induce STAT3 Activation in Primary Neurons and SH-SY5Y Cells. A , Primary neurons were treated with IL-6 (10 ng/ml) plus sIL-6R (25 ng/ml) or OSM (10 ng/ml) for the times indicated. Protein lysates were prepared and subjected to immunoblot analyses with antibodies against phosphorylated STAT3 Tyr705, phosphorylated STAT3 Ser727, and total STAT3. **B**, SH-SY5Y cells were treated with IL-6 (10 ng/ml) plus sIL-6R (25 ng/ml) or OSM (10 ng/ml) for the times indicated, and cell lysates immunoblotted as described. The densitometric ratios of P-STAT3 Tyr705 or P-STAT3 Ser727 versus total STAT3 were calculated, and are shown as Fold Increase. Graphs represent the mean ± SEM for triplicate cultures in three separate experiments. *p<0.001, #p<0.01, and **p<0.05 compared to control, untreated cultures.

### IL-6 Cytokines Induce STAT3 Activation in Primary Neurons and SH-SY5Y Cells

Gp130 is the common signal transducing receptor subunit for all IL-6 cytokine family members [1], [32]. Binding of IL-6 cytokines to their specific receptors induces activation of the JAK/STAT signaling pathway, particularly STAT3 [1]. Thus, we investigated whether IL-6 cytokines induce phosphorylation of STAT3 in neuron-enriched cortical cultures. We focused on IL-6/R and OSM for the remainder of the study, as CNTF was not protective in primary neurons and little is known regarding IL-11 expression *in vivo*. Immunoblotting analyses of primary neurons demonstrated that IL-6/R and OSM induced tyrosine phosphorylation of STAT3 (P-STAT3 Tyr) within 30 min, which was maintained up to 4 h (Figure 2A). Basal levels of serine phosphorylated STAT3 (P-STAT3 Ser) were observed in primary neurons, which were enhanced by IL-6/R at 30 min and 1 h, and at 30 min by OSM in primary neurons. In SH-SY5Y cells, tyrosine and serine phosphorylation of STAT3 was detected 30 min after treatment with IL-6/R or OSM, and maintained for 4-8 h (Figure 2B). Immunoblot analyses demonstrate moderate changes in the levels of total STAT3 in response to cytokine stimulations, perhaps due to changes in the phosphorylation status. These results indicate that IL-6/R and OSM can promote activation of the STAT3 transcription factor in neurons.

**Figure 3:**
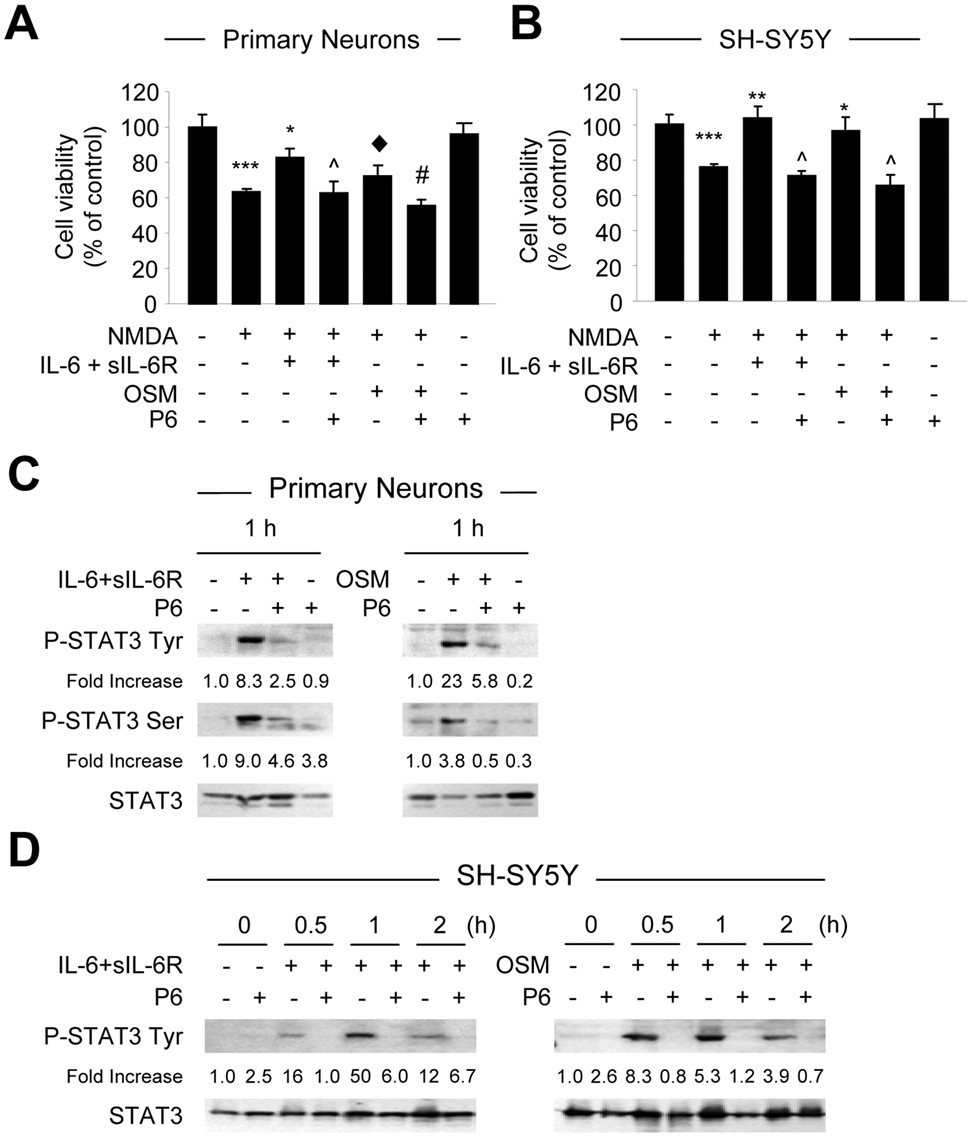
P6 Reverses the Protective Effect of IL-6 Cytokines on NMDA-induced Toxicity. **A**–**B**, Primary cortical neurons (A) or SH-SY5Y cells (B) were pretreated with P6 (0.5 µM) for 1 h, and then grown in the presence of P6 and IL-6 plus sIL-6R or OSM for 1 h, and then in the presence of cytokine, P6, plus NMDA for an additional 24 h. Cell viability was determined by the MTT reduction assay. Graphic representation of the mean ± SEM of triplicate cultures in three separate experiments. ***p<0.001 compared to control; **p<0.001, *p<0.01, and ♦p<0.05 compared to NMDA; and ?p<0.001 and #p<0.01 compared to NMDA plus cytokines. **C**–**D**, P6 Inhibits IL-6 Cytokine-induced STAT3 Activation. Primary neurons (C) or SH-SY5Y cells (D) were grown with P6 for 1 h and where indicated, grown in the presence of OSM or IL-6 plus sIL-6R and P6. The levels of phosphorylated STAT3 Tyr705, phosphorylated STAT3 Ser727, and total STAT3 were analyzed at the indicated time points. The densitometric ratios of P-STAT3 Tyr705 or P-STAT3 Ser727 versus total STAT3 were calculated, and shown as Fold Increase. Graph represents the mean ± SEM of triplicate cultures in four separate experiments. *p<0.001 and **p<0.01 compared to control; and ?p<0.001, #p<0.01, and &p<0.05 compared to IL-6 plus IL-6R or OSM alone.

### P6 Reverses the Protective Effect of IL-6 Cytokines on NMDA-induced Toxicity

We hypothesized that the protective effect of IL-6/R and OSM against NMDA-induced neuronal death was mediated in part by STAT3 activation. To test this, we investigated whether inhibition of JAK/STAT signaling by P6, a pan-JAK inhibitor [22], altered the protective effect of IL-6/R and OSM on NMDA-induced toxicity. Pretreatment of P6 for 1 h and continued exposure for 24 h in the presence of NMDA did not affect NMDA-induced toxicity of primary neurons (Figure 3A) or SH-SY5Y cells (Figure 3B). However, pretreatment with P6 reversed the protective effect of IL-6/R and OSM on NMDA-induced neuronal death by 20% and 17% in cortical neurons (Figure 3A) and by 21% and 30% in SH-SY5Y cells (Figure 3B). Next, we tested whether P6 treatment inhibited IL-6/R and OSM-induced STAT3 phosphorylation. Immunoblotting analyses demonstrated that a 1 h pretreatment with P6 and continued P6 exposure plus cytokine for indicated time points inhibited IL-6/R and OSM-induced tyrosine and serine STAT3 phosphorylation in primary neurons and SH-SY5Y cells (Figures 3C–D). Additional experiments to investigate the effects of NMDA on STAT3 activation demonstrated that NMDA alone does not induce STAT3 activation, nor does it significantly impact cytokine-induced STAT3 activation (Figure S2).

**Figure 4:**
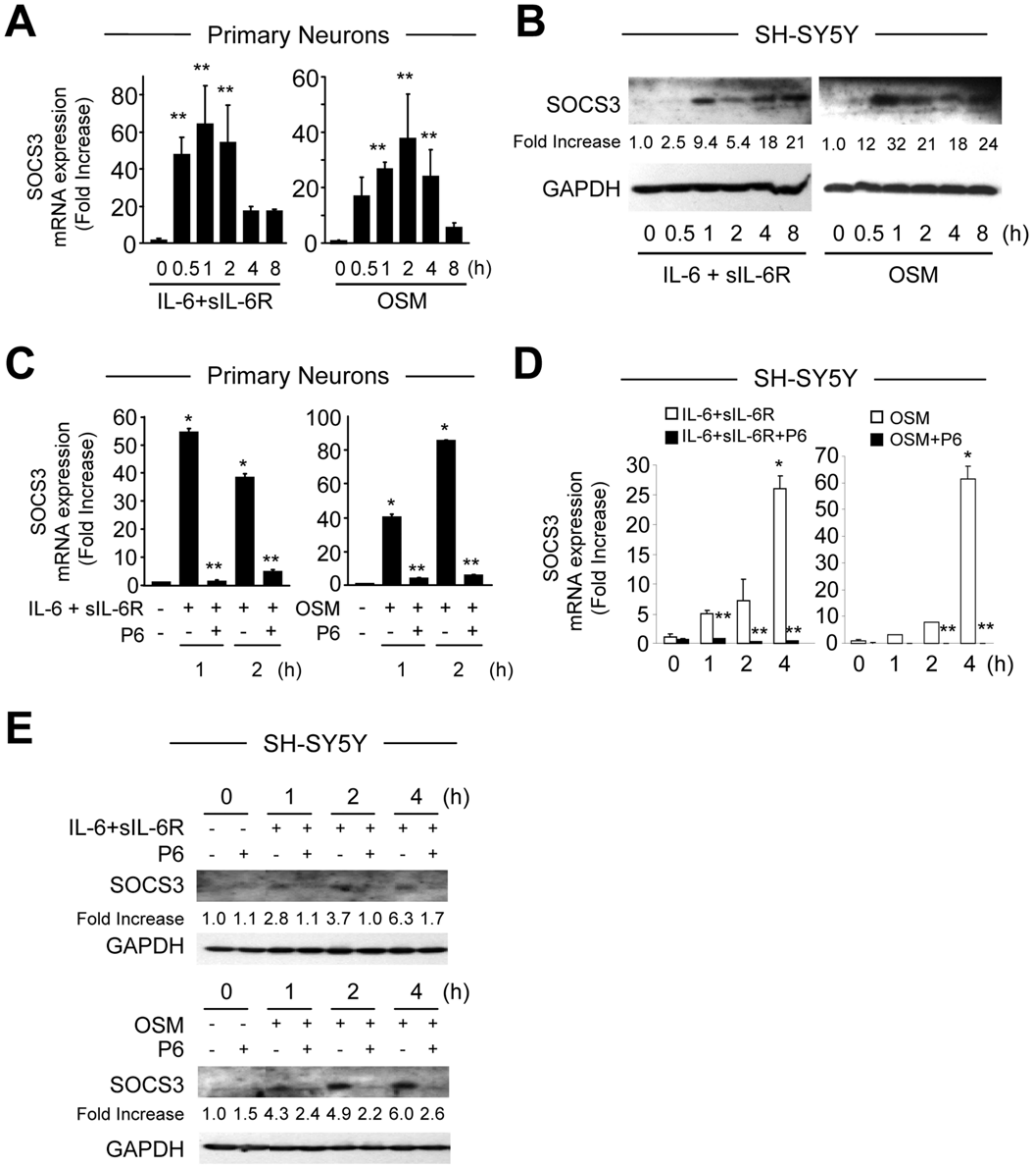
Induction of SOCS3 Expression by IL-6 Cytokines in Primary Neurons and SH-SY5Y Cells. **A,** Primary neurons were treated with IL-6 (10 ng/ml) plus sIL-6R (25 ng/ml) or OSM (10 ng/ml) for the times indicated, and total RNA was analyzed by qRT-PCR. Graphic representation of the mean ± SEM of triplicate cultures in three separate experiments. **p<0.001 compared to control. **B**, SH-SY5Y cells were treated with IL-6 (10 ng/ml) plus sIL-6R (25 ng/ml) or OSM (10 ng/ml) for the times indicated, and cell lysates immunoblotted with SOCS3 and GAPDH antibodies. **C**–**E,** Inhibition of the JAK/STAT Pathway Inhibits SOCS3 Expression. **C, D**, Primary cortical neurons (C) or SH-SY5Y cells (D) were pretreated with P6 (0.5 µM) for 1 h, and then treated with IL-6 plus sIL-6R or OSM in the presence of continued P6 exposure, and total mRNA was analyzed by qRT-PCR. Graphic representation of the mean ± SEM of triplicate cultures in three separate experiments. *p<0.001 compared to control; **p<0.001 compared to IL-6 plus IL-6R or OSM alone. **E**, SH-SY5Y cells were grown in the absence or presence of P6 pretreatment for 1 h and then grown in the presence of P6 and IL-6 plus sIL-6R or OSM and protein levels of SOCS3 were analyzed. The densitometric ratios of SOCS3 versus GAPDH were calculated. Graph represents the mean ± SEM of triplicate cultures in three separate experiments. *p<0.001 compared to control; ?p<0.001 compared to IL-6 plus IL-6R or OSM alone.

### JAK/STAT3 Activation is Necessary for SOCS3 Expression in Neurons

Negative regulation of the JAK/STAT pathway is necessary for appropriate physiological responses to cytokine stimulation [33]. SOCS3 expression in some cell types is induced by treatment with cytokines of the IL-6 family via STAT3 activation [13], [26], indicating that SOCS3 is a STAT3-inducible gene. We tested whether SOCS3 expression was regulated upon IL-6/R or OSM treatment in neurons. Basal expression levels of SOCS3 mRNA were very low in untreated primary neurons. However, IL-6/R or OSM-induced SOCS3 mRNA expression was detectable at 30 min, peaked at 1–2 h, and declined by 8 h (Figure 4A). SOCS3 protein expression was detected between 1–8 h in SH-SY5Y cells upon IL-6/R or OSM treatment (Figure 4B). To evaluate whether SOCS3 expression requires JAK/STAT3 activation, primary cortical neurons (Figure 4C) or SH-SY5Y cells (Figures 4D, E) were pretreated for 1 h with P6, and then continually exposed to P6 plus cytokine for indicated time points. The JAK inhibitor P6 significantly inhibited IL-6/R or OSM-induced SOCS3 mRNA and protein expression at all time points examined, indicating that activation of the JAK/STAT3 pathway is necessary for subsequent SOCS3 expression.

**Figure 5:**
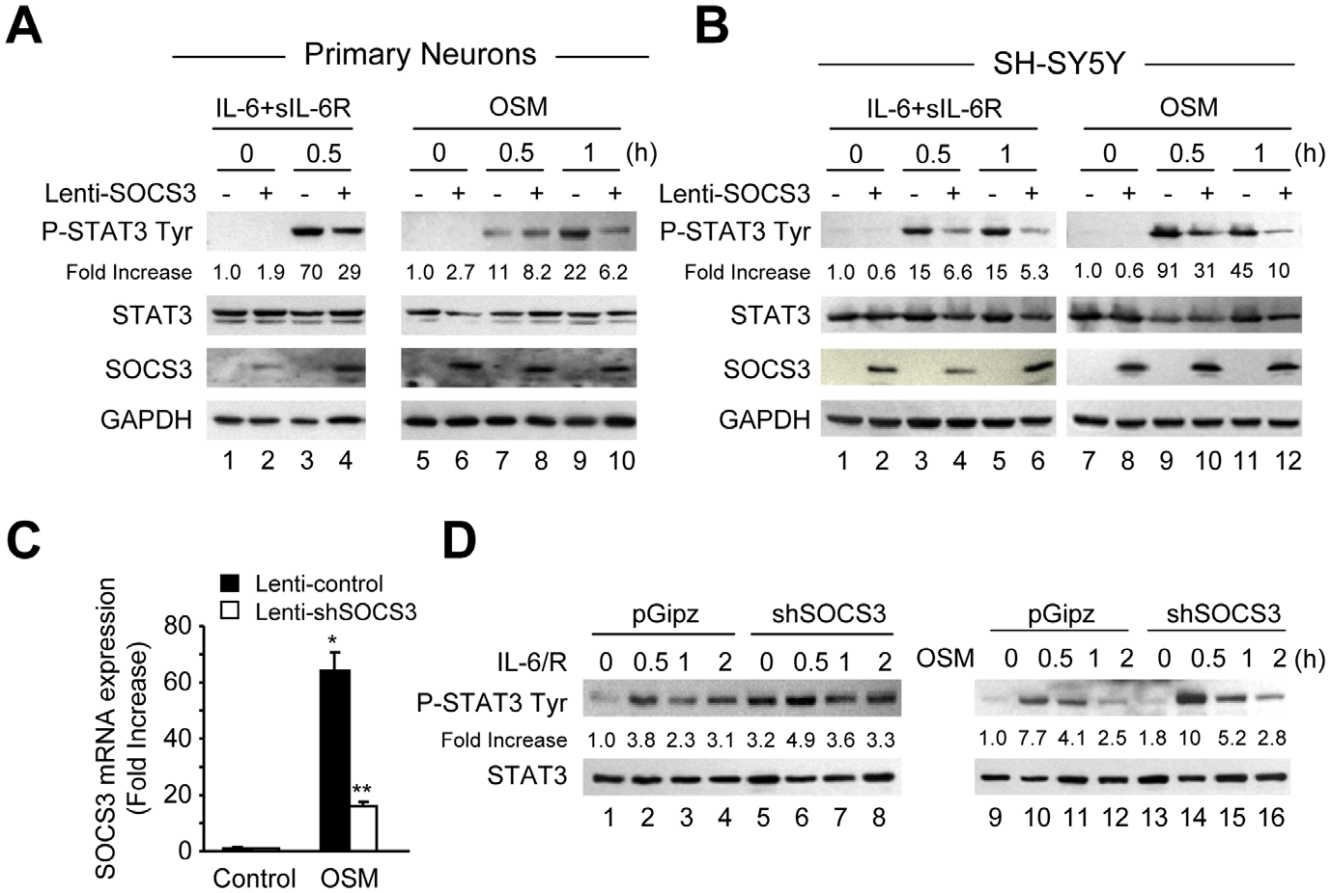
SOCS3 Inhibits STAT3 Activation in Primary Neurons and SH-SY5Y Cells. **A**–**B**, Primary neurons (A) or SH-SY5Y cells (B) were infected with Lenti-control (−) or Lenti-SOCS3 (+). After infection, IL-6 plus sIL-6R or OSM was added to primary cortical neurons or SH-SY5Y cells for up to 1 h, and protein lysates were subjected to immunoblot analysis with antibodies against phosphorylated STAT3 Tyr705, total STAT3, SOCS3 and GAPDH. The densitometric ratios of P-STAT3 Tyr705 versus total STAT3 were calculated, and shown as Fold Increase. Graph represents the mean ± SEM of triplicate cultures in three separate experiments. *p<0.001 compared to Lenti-control infected cells left untreated; ?p<0.001, and ??p<0.01 compared to Lenti-control infected cells treated with IL-6 plus IL-6R or OSM. **C**, SH-SY5Y cells were infected with control or shSOCS3 lentivirus, treated with OSM for 1 h, and total RNA was analyzed by qRT-PCR for SOCS3 expression. Graphic representation of the mean ± SEM of triplicate cultures in three separate experiments. *p<0.001 compared to control; **p<0.001 compared to OSM treatment of control lentivirus infected culture. **D**, After infection of SH-SY5Y cells with pGipz (control) or shSOCS3 lentivirus, IL-6 plus sIL-6R or OSM was added for the times indicated and cell lysates immunoblotted as described. The densitometric ratios of P-STAT3 Tyr705 versus total STAT3 were calculated, and shown as Fold Increase. Graph represents the mean ± SEM of triplicate cultures in three separate experiments. *p<0.001 compared to control cultures infected with pGipz; ?p<0.001, and ??p<0.01 compared to IL-6 plus IL-6R or OSM treatment of pGipz lentivirus infected cultures.

### SOCS3 Inhibits IL-6 Cytokine-induced STAT3 Activation and Protective Effect on Neurons

We next investigated whether SOCS3 regulates IL-6 cytokine induced-STAT3 activation in neurons. Primary neurons (Figure 5A) or SH-SY5Y cells (Figure 5B) were infected with SOCS3 (Lenti-SOCS3) or GFP as a control (Lenti-control), and then treated with IL-6/R or OSM. Primary neurons infected with Lenti-control treated with IL-6/R or OSM induced STAT3 phosphorylation (Figure 5A, lanes 3, 7 and 9). However, expression of exogenous SOCS3 inhibited cytokine induced-STAT3 tyrosine phosphorylation in primary neurons (Figure 5A, lanes 4, 8 and 10). Comparable results were obtained with SH-SY5Y cells (Figure 5B). To further confirm negative regulation by SOCS3 on IL-6 cytokine-induced STAT3 activation, SH-SY5Y cells were infected with lentivirus expressing shRNA targeting endogenous SOCS3 (Lenti-shSOCS3) or control lentivirus (pGipz), and then treated for 1 h with OSM. qRT-PCR analysis showed that shSOCS3 expression led to significant inhibition of OSM-induced SOCS3 mRNA expression (Figure 5C). Next, SH-SY5Y cells were infected with Lenti-shSOCS3 or pGipz and treated with IL-6/R or OSM. We observed that shSOCS3 expression enhanced both basal (Figure 5D, compare lanes 1 and 5; lanes 9 and 13) and cytokine induced-STAT3 phosphorylation (compare lanes 2–4 with lanes 6–8; and lanes 10–12 with lanes 14–16), compared to cells exposed to pGipz (Figure 5D).

**Figure 6:**
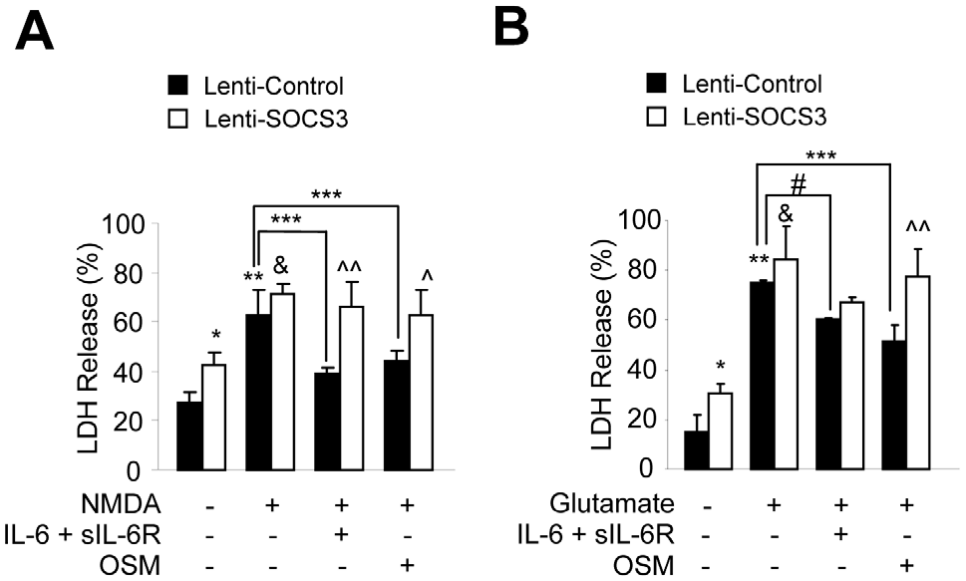
SOCS3 Abrogates the Protective Effect of IL-6 Cytokines on NMDA and Glutamate-induced Toxicity. **A**–**B**, Primary neurons were infected with Lenti-control or Lenti-SOCS3. After infection, cells were stimulated with IL-6 plus sIL-6R or OSM for 1 h, and then grown in the presence of cytokine and NMDA (100 µM, **A**) or glutamate (100 µM, **B**) for an additional 24 h. Cell viability was determined by the LDH release assay. Graphic representation of the mean ± SEM of triplicate cultures in three separate experiments. *p<0.05 and **p<0.001 compared to control lentivirus infected cultures; &p<0.001 compared to SOCS3 lentivirus infected cultures; #p<0.05 and ***p<0.001 compared to NMDA/glutamate treatment of control lentivirus infected cultures; and ?p<0.01 and ??p<0.001 compared to each cytokine plus NMDA or glutamate treatment of control lentivirus infected cells.

We next determined whether SOCS3 expression influenced the protective effect of IL-6 cytokines on neuronal death. Primary neurons were infected with Lenti-control or Lenti-SOCS3 and cells were pretreated with IL-6/R or OSM for 1 h, and then grown in the presence of cytokine and NMDA or glutamate for an additional 24 h. Results from cell viability assays demonstrated that the protective effect of IL-6/R or OSM on NMDA-induced neuronal death was inhibited upon expression of SOCS3 (Figure 6A), and this SOCS3-mediated effect was also observed in glutamate-treated cultures (Figure 6B).

**Figure 7:**
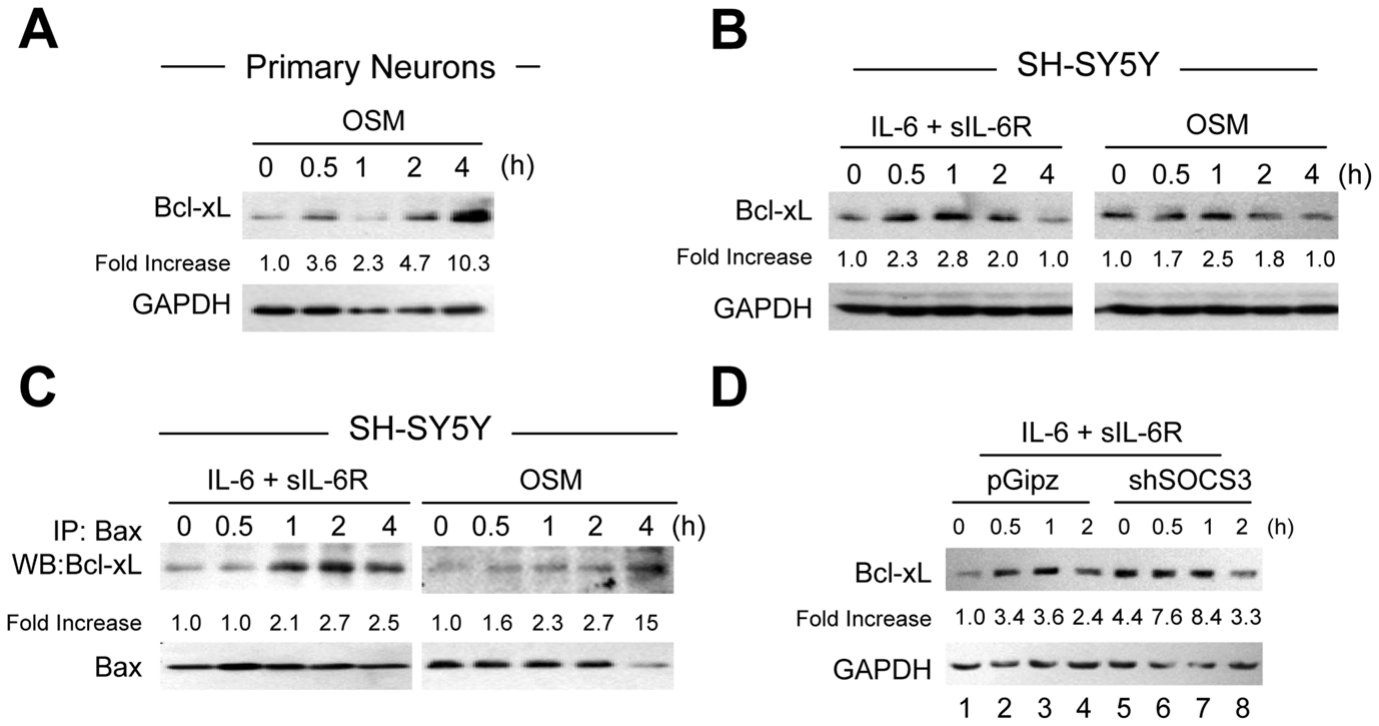
Bcl-xL Expression is Regulated by IL-6 Cytokines and SOCS3. **A**–**B,** Primary neurons (A) were treated with OSM (10 ng/ml) for the times indicated, or SH-SY5Y cells (B) were treated with IL-6 (10 ng/ml) plus sIL-6R (25 ng/ml) or OSM (10 ng/ml) for the times indicated. Protein lysates were prepared and subjected to immunoblot analyses with antibodies against Bcl-xL and GAPDH. The densitometric ratios of Bcl-xL versus GAPDH were calculated. Graph represents the mean ± SEM of triplicate cultures in three separate experiments. *p<0.001, and **p<0.01 compared to control. **C**. Co-IP of Bax with Bcl-xL. Lysates from each sample were subjected to immunoprecipitation (IP) using antibody specific for Bax. IP samples were probed with antibody to Bcl-xL to demonstrate a specific interation between Bcl-xL and Bax. The densitometric ratios of co-immunoprecipitated Bcl-xL to immunoprecipitated Bax were calculated. Graph represents the mean ± SEM of triplicate cultures in three separate experiments. *p<0.001, **p<0.01, and ***p<0.05 compared to control. **D**, SH-SY5Y cells were infected with pGipz or Lenti-shSOCS3. After infection, IL-6 plus sIL-6R was added for the times indicated, and cell lysates immunoblotted as described. The densitometric ratios of Bcl-xL versus GAPDH were calculated. Graph represents the mean ± SEM of triplicate cultures in three separate experiments. *p<0.001, and **p<0.01 compared to pGipz-infected cultures; and &p<0.01 compared to IL-6 plus IL-6R treatment of pGipz-infected cultures.

### SOCS3 Inhibits Bcl-xL Expression

STAT3 activation initiates transcription of genes involved in neuronal cell survival such as Bcl-xL and Bcl-2 [5]; as such, we examined whether IL-6/R or OSM-induced STAT3 regulated Bcl-xL expression in neurons. Immunoblotting analyses demonstrated basal expression of Bcl-xL in untreated cultures, and OSM increased Bcl-xL expression in a time-dependent manner in both primary neurons (Figure 7A) and in SH-SY5Y cells (Figure 7B). As Bcl-xL inhibits cell death by binding Bax, which promotes cell death [34], [35], we investigated whether IL-6 cytokine-increased Bcl-xL binds to Bax. SH-SY5Y cells were treated with IL-6/R or OSM for the indicated times and co-immunoprecipitation experiments were performed. The results demonstrate that Bcl-xL binds to Bax, and this association is enhanced in response to IL-6/R or OSM in a time-dependent manner (Figure 7C). We next tested whether SOCS3 regulates Bcl-xL expression. SH-SY5Y cells were infected with Lenti-shSOCS3 or pGipz and then treated with IL-6/R. We observed that reduced SOCS3 expression by Lenti-shSOCS3 enhanced basal expression of Bcl-xL expression (Figure 7D, lanes 1 and 5), and enhanced IL-6/R up-regulation of Bcl-xL, compared to pGipz infected cells (lanes 2–4 compared to lanes 6–8). These results indicate that SOCS3 is a negative regulator of Bcl-xL expression.

**Figure 8:**
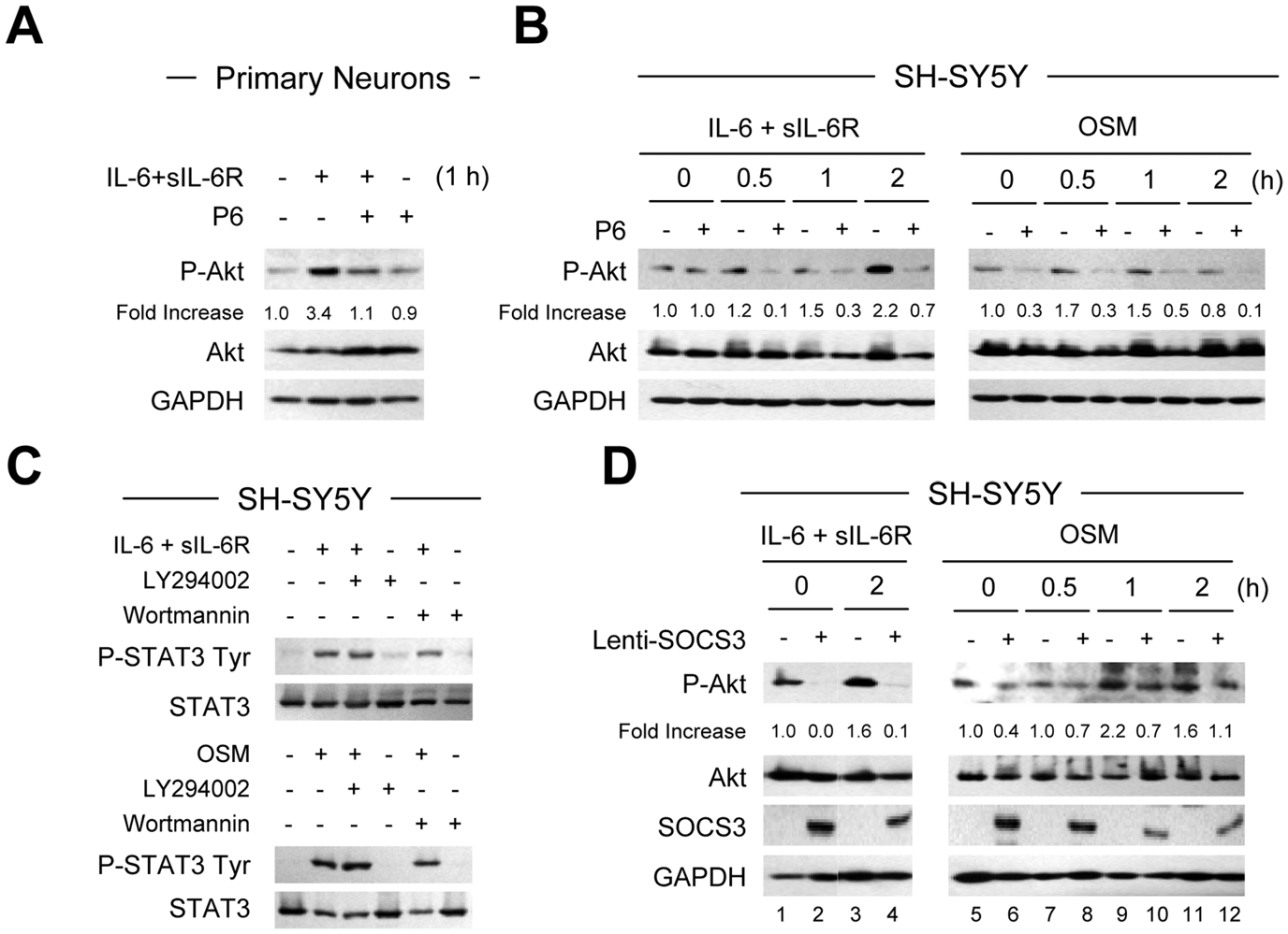
Regulation of the Akt Signaling Pathway by IL-6 Cytokines and SOCS3. **A**–**B**, Primary neurons (A) were treated with IL-6 (10 ng/ml) plus sIL-6R (25 ng/ml), or SH-SY5Y cells (B) were treated with IL-6 (10 ng/ml) plus sIL-6R (25 ng/ml) or OSM (10 ng/ml) in the absence or presence of P6 (0.5 µM) pretreatment for 1 h, and protein lysates subjected to immunoblot analyses with antibodies against P-Akt, total Akt and GAPDH. P6 was also present for indicated time points of cytokine exposure. The densitometric ratios of P-Akt versus total Akt were calculated, and shown as Fold Increase. Graph represents the mean ± SEM of triplicate cultures in three separate experiments. *p<0.001, **p<0.01, and ***p<0.05 compared to control; ?p<0.001, &p<0.01, and ??p<0.05 compared to IL-6 plus IL-6R or OSM alone. **C**, SH-SY5Y cells were pretreated for 2 h with the PI3-Kinase inhibitors LY294002 (10 µM) or Wortmannin (1 µM), followed by treatment with IL-6 plus sIL-6R or OSM for 30 min with continued LY294002 or Wortmannin exposure, and protein levels of phosphorylated STAT3 Tyr705 and total STAT3 were analyzed. The densitometric ratios of P-STAT3 Tyr705 versus total STAT3 were calculated, and shown as Fold Increase. Graph represents the mean ± SEM of triplicate cultures in three separate experiments. *p<0.001 compared to control. **D**, SH-SY5Y cells were infected with Lenti-control (−) or Lenti-SOCS3 (+). After infection, IL-6 plus sIL-6R or OSM was added to SH-SY5Y cells for the times indicated, and protein lysates subjected to immunoblot analyses with antibodies against P-Akt, total Akt, SOCS3 and GAPDH. The densitometric ratios of P-Akt versus total Akt were calculated, and shown as Fold Increase. Graph represents the mean ± SEM of triplicate cultures in three separate experiments. *p<0.001 compared to Lenti-control lentivirus infected cultures left untreated; ?p<0.001 compared to IL-6 plus IL-6R or OSM treatment of control lentivirus infected cells.

### SOCS3 Regulates the Akt Signaling Pathway

Previous studies have demonstrated that the anti-apoptotic effect of IL-6 depends on PI3-Kinase, which is upstream of the Akt pathway [36], [37]. It has also been shown that the protective effect of STAT3 on hippocampal neurons is inhibited by the PI3-Kinase inhibitors LY294002 or wortmannin [5], suggesting cross-talk between the STAT3 and PI3-Kinase/Akt pathways. We examined the influence of IL-6 cytokines on phosphorylation/activation of Akt in neurons. Immunoblotting analyses demonstrated that basal Akt phosphorylation was detected in cultures of primary neurons and SH-SY5Y cells (Figures 8A and 8B). Treatment with IL-6/R increased Akt phosphorylation in primary neurons (Figure 8A) and IL-6/R and OSM enhanced Akt phosphorylation in SH-SY5Y cells (Figure 8B). However, pretreatment with P6 for 1 h and then continuous exposure to P6 plus cytokine treatment demonstrated reduced IL-6/R and OSM-enhanced Akt phosphorylation (Figures 8A, B), indicating that the JAK/STAT3 signaling pathway is upstream of Akt. Inclusion of the PI3-Kinase inhibitors LY294002 or Wortmannin did not affect IL-6/R or OSM-induced activation of the STAT3 pathway (Figure 8C). To investigate whether SOCS3 regulates the Akt signaling pathway, SH-SY5Y cells were infected with Lenti-SOCS3 or Lenti-control, and then treated with cytokines. Lenti-control infected cells treated with IL-6/R or OSM demonstrated increased Akt phosphorylation (Figure 8D), and expression of Lenti-SOCS3 strongly inhibited IL-6/R or OSM-induced Akt phosphorylation in SH-SY5Y cells.

**Figure 9:**
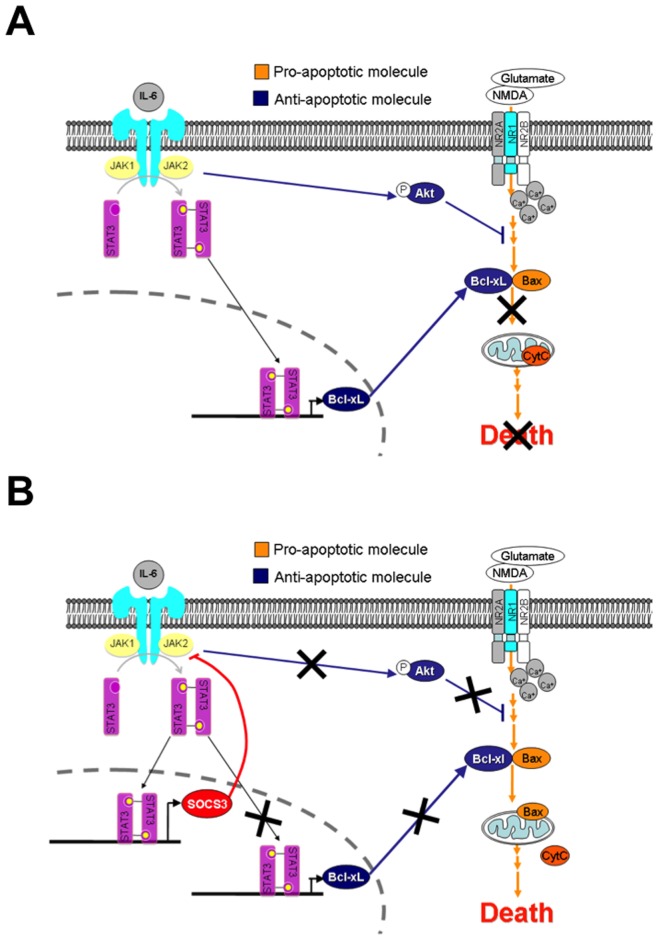
Proposed Model for SOCS3 Contribution to Excitoxicity in Neurons. A , IL-6/R or OSM activates the JAK/STAT3 pathway, which increases Bcl-xL expression and causes activation of the Akt signaling pathway in a STAT3-dependent manner. Increased Bcl-xL expression and binding to Bax, as well as Akt activation inhibits NMDA or glutamate-induced neuronal death. **B**, IL-6/R and OSM also induce SOCS3 expression in a STAT3-dependent fashion. SOCS3, in turn, modulates IL-6/R or OSM induced Bcl-xL expression and Akt signaling pathway in a negative regulatory manner, which contributes, in part, to NMDA or glutamate-induced neuronal death.

## Discussion

We demonstrate the expression of SOCS3 in neurons in response to IL-6 cytokines, and the negative regulatory effect of SOCS3 on STAT3 activation, which consequently contributes to excitotoxic neuronal death *in vitro*. IL-6 cytokine treatment induces STAT3 activation in primary neurons and SH-SY5Y cells, which also promotes SOCS3 expression as a transcriptional target gene. Results from experimental strategies to overexpress exogenous SOCS3 as well as inhibit endogenous SOCS3 expression demonstrate that SOCS3 functions as a negative regulator of activated STAT3, which subsequently inhibits the protective effect of activated STAT3 on NMDA or glutamate-induced neurotoxicity. Lastly, IL-6 cytokine-induced Bcl-xL expression and activation of the Akt pathway is negatively impacted by SOCS3 expression, supporting the concept that SOCS3 contributes to neuronal death. We note that in primary neurons, IL-6, OSM and IL-11 had protective effects, while CNTF did not. CNTF signaling is complex, involving membrane-bound or soluble CNTFRα, as well as the LIF receptor [38]. It may be that the appropriate composition of CNTFRα, LIFR and gp130 is not present on primary neurons to mediate a protective effect by CNTF.

Activated STAT3 functions as a key effector of neuronal survival after injury to neural tissue, in part by induction of anti-apoptotic genes such as Bcl-xL, Bcl-2 and Mn-SOD [4], [39]. In an *in vivo* ischemic model, estradiol-induced activation of STAT3 in neurons reduced MCAO-induced infarct size and induced Bcl-2 expression, thereby producing a neuroprotective effect [40]. Our current study demonstrates that IL-6 cytokine-induced phosphorylation/activation of STAT3 inhibits NMDA or glutamate-induced toxicity. Moreover, this protective effect of STAT3 was reversed by P6, a pan-JAK inhibitor, indicating that IL-6 cytokine-induced activation of the JAK/STAT pathway promotes survival of neurons under excitotoxic conditions. One of the underlying mechanisms of the IL-6 cytokine-induced STAT3 protective effect is increased expression of Bcl-xL, and Bcl-xL binding to Bax, which then promotes neuronal survival. Our results are consistent with recent reports showing that leptin-induced STAT3 protects against excitotoxicity through regulation of Bcl-xL expression in rat hippocampal neurons [5]. Collectively, these results indicate that IL-6 cytokine-induced STAT3 activation promotes neuronal survival in part by inducing the expression of the anti-apoptotic gene, Bcl-xL.

Several studies demonstrate that the anti-apoptotic effect of IL-6 cytokines depends on activation of the JAK/STAT3 pathway, as well as PI3-Kinase activation, which is upstream of the Akt pathway [36]. The protective effect of activated STAT3 on hippocampal neurons is inhibited by the PI3-Kinase inhibitors, LY294002 or wortmannin [5]. In our study, the pan-JAK inhibitor P6 suppressed IL-6 cytokine-induced Akt phosphorylation in neurons, whereas PI3-kinase inhibitors had no effect on STAT3 activation, indicating that the JAK/STAT3 pathway is upstream of PI3-Kinase/Akt.

The major function of SOCS3 is to limit signaling by the IL-6 family of cytokines by inhibiting JAK tyrosine kinase activity, thereby preventing STAT3 activation [41], [42]. As SOCS3 deficiency is embryonic lethal [43], conditional gene targeting has been used to elucidate the function of SOCS3. Targeted deletion of SOCS3 in mouse brain and cultured neurons results in elevated STAT3 activation as induced by CNTF and leptin treatment [10], [44]–[46]. Our results indicate that in neurons, SOCS3 functions as a negative regulator of IL-6/R or OSM induced-JAK/STAT3 activation and downstream signaling pathways, including Akt. Expression of exogenous SOCS3 inhibited IL-6/R or OSM-induced STAT3 phosphorylation in neurons, while reduction in endogenous SOCS3 led to enhanced cytokine induced-STAT3 phosphorylation. SOCS3 expression in neurons plays a negative role in regulation of cell survival and neurite outgrowth [20], [21], [47], [48]. In dorsal root ganglion neurons, over-expression of SOCS3 by lentivirus inhibited neurite outgrowth, whereas inhibition of endogenous SOCS3 expression enhanced neurite outgrowth [10]. Smith et al., [21] reported that in optic nerve, conditional deletion of SOCS3 promoted regeneration of injured optic nerve axons. In SOCS3-gp130 double knockout mice, the regeneration effect was ablated, suggesting that SOCS3 deletion regulates optic nerve regeneration via an gp130-dependent pathway [21]. In our study, reducing endogenous SOCS3 expression in neurons enhanced STAT3 activation and IL-6 cytokine-induced Bcl-xL expression. It has recently been reported that neuronal SOCS3 inhibits the PI3-Kinase pathway in mouse hypothalamus and neuronal cell lines [49], and that deletion of SOCS3 enhanced leptin-induced PI3-Kinase activity *in vivo*. *In vitro,* leptin-induced activation of the PI3-Kinase pathway involved activation of JAK2 [49]. Our results indicate that over-expression of SOCS3 inhibits IL-6 cytokine-induced Akt phosphorylation. Additionally, we demonstrated that STAT3 activation is upstream of the PI3-Kinase/Akt pathway. Collectively, these findings suggest that binding of SOCS3 directly to JAK2 may negatively regulate IL-6 cytokine-induced activation of the PI3-Kinase pathway. Other SOCS proteins may be involved in this negative regulatory pathway such as SOCS1 and SOCS2, but SOCS3 is the most effective inhibitor of STAT3 activation [14].

We propose a two-step model for how activated STAT3 can enhance neuronal growth/survival, as well as contribute to neurotoxicity (Figure 9). Injury to both the CNS and PNS leads to the rapid expression of IL-6 cytokines in lesion sites. Elevated IL-6 expression is associated with STAT3 phosphorylation/activation, and neuronal growth/axonal regeneration (Figure 9A). Aspects of this beneficial STAT3 activation include induction of anti-apoptotic proteins (Bcl-xL), activation of the pro-survival PI3-Kinase/Akt signaling pathway (P-Akt), and induction of a neuronal growth program [4], [12], [50], [51]. However, STAT3 activation also leads to induction of SOCS3, which functions to limit the JAK/STAT pathway (Figure 9B). The functional consequences of SOCS3 expression include inhibition of STAT3 activation, inhibition of Bcl-xL expression, and suppression of the pro-survival Akt signaling pathway, all of which can contribute to the loss of the protective effects of IL-6 cytokines on neuronal survival. The elegant studies of Smith el al., [21] and Sun et al., [48] convincingly demonstrate that upon conditional deletion of SOCS3, extensive axonal regeneration occurs after optic nerve injury. Furthermore, administration of CNTF to SOCS3-deleted mice significantly increased the extent of axon regeneration, compared to mice with an intact SOCS3 gene [21]. These findings collectively demonstrate the two faces of the JAK/STAT3 pathway; the regenerative capacity of activated STAT3, and the subsequent induction of SOCS3, which then functions as a negative regulator, restricting responsiveness to IL-6 cytokines and neuronal survival.

The function of SOCS3 in the brain is cell type specific and complex [13]. Previous studies from our laboratory indicate that in microglia, SOCS3 attenuates cytokine-induced immune and inflammatory responses [52], [53] and in astrocytes SOCS3 exerts an inhibitory effect on chemokine expression and T-cell migration [25]. Early activation of JAK/STAT3 signaling in the spinal cord is thought to contribute to the development of neuroinflammatory responses and mechanical allodynia [54]. Lentiviral-mediated SOCS3 expression predominantly in microglia blocked spinal cord neuroinflammation and attenuated the development of mechanical allodynia [54]. Further, SOCS3 expression in macrophages, microglia and dendritic cells is critical for deactivation of neuroinflammatory responses in an animal model of Multiple Sclerosis [55], [56]. Thus, SOCS3 expression in macrophages, microglia and astrocytes suppresses brain inflammation. However, the role of SOCS3 in neurons appears to be detrimental by inhibiting STAT3 activation and downstream neuroprotective effects. A similar situation exists for oligodendrocytes. LIF and CNTF function as survival factors for oligodendrocytes [57]–[59]. However, LIF and CNTF induce SOCS3 expression in oligodendrocytes, which dampens their protective effects. In this regard, mice lacking SOCS3 expression in oligodendrocytes display less oligodendrocyte loss after cuprizone insult compared with wild-type mice [60]. Thus, as SOCS3 has vastly different functions, dependent on cell-type specific expression, it is critical to understand how SOCS3 expression is both induced and repressed. SOCS3 may indeed be a therapeutic target in a number of neurologic diseases, however, strategies to selectively inhibit SOCS3 in neurons and oligodendrocytes, or induce SOCS3 in microglia and astrocytes, will need to be explored.

## Supporting Information

Figure S1 **Purity of Mouse Primary Cortical Neuron Cultures. A**–**B**, Primary neurons derived from embryonic day 17 C57BL/6J mice were grown on glass coverslips for 11 days *in vitro* (DIV) and immunostained with MAP-2 for neurons (A) and GFAP for astrocytes (B). Immunostained cells were subjected to bright-field microscopy. **C**, Primary neurons from four different cultures at DIV 11 were collected, lysed and subjected to immunoblot analyses with antibody against MAP-2.
(TIFF)

Figure S2 **NMDA Does Not Affect STAT3 Activation in SH-SY5Y Cells.** SH-SY5Y cells were pretreated for 1 h with OSM, followed by co-exposure of cytokine with NMDA (1 mM) for 5 and 10 min, and protein levels of phosphorylated STAT3 Tyr705 and total STAT3 were analyzed. The densitometric ratios of *P-STAT3 Tyr705* versus *total STAT3* were calculated, and shown as Fold Increase. Graph represents the mean ± SEM of triplicate cultures in three separate experiments. *p<0.001 compared to untreated cultures.
(TIFF)
