# Supplementary figures and images for: Protective Role of STAT3 in NMDA and Glutamate-Induced Neuronal Death: Negative Regulatory Effect of SOCS3
Source: PLoS One. 2012 Nov 30;7(11):e50874. doi: 10.1371/journal.pone.0050874 (PMC3511325; doi:10.1371/journal.pone.0050874)

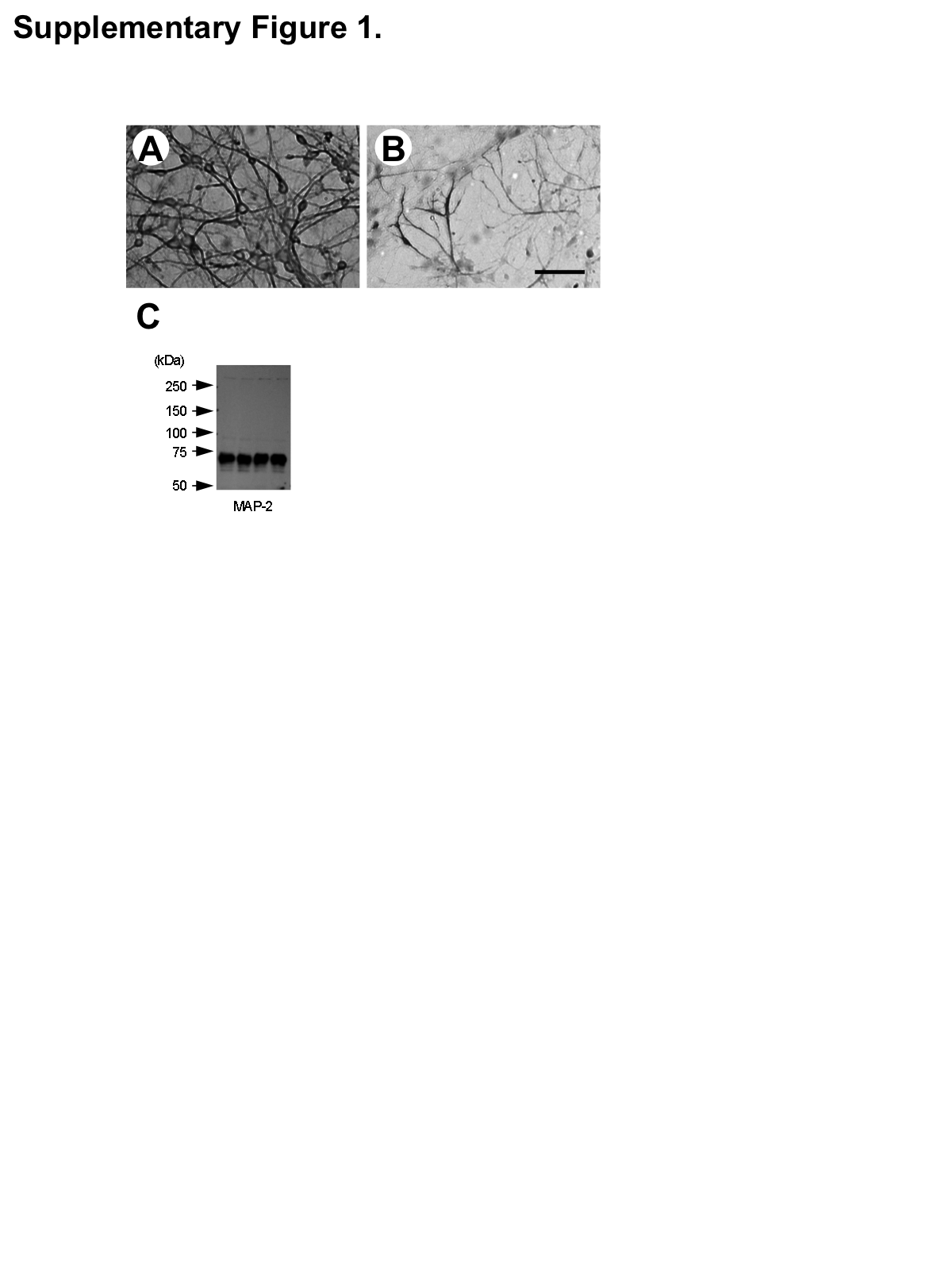

Supplement: Figure S1 — Purity of Mouse Primary Cortical Neuron Cultures. A–B, Primary neurons derived from embryonic day 17 C57BL/6J mice were grown on glass coverslips for 11 days in vitro (DIV) and immunostained with MAP-2 for neurons (A) and GFAP for astrocytes (B). Immunostained cells were subjected to bright-field microscopy. C, Primary neurons from four different cultures at DIV 11 were collected, lysed and subjected to immunoblot analyses with antibody against MAP-2. (TIFF) [file pone.0050874.s001.tif]

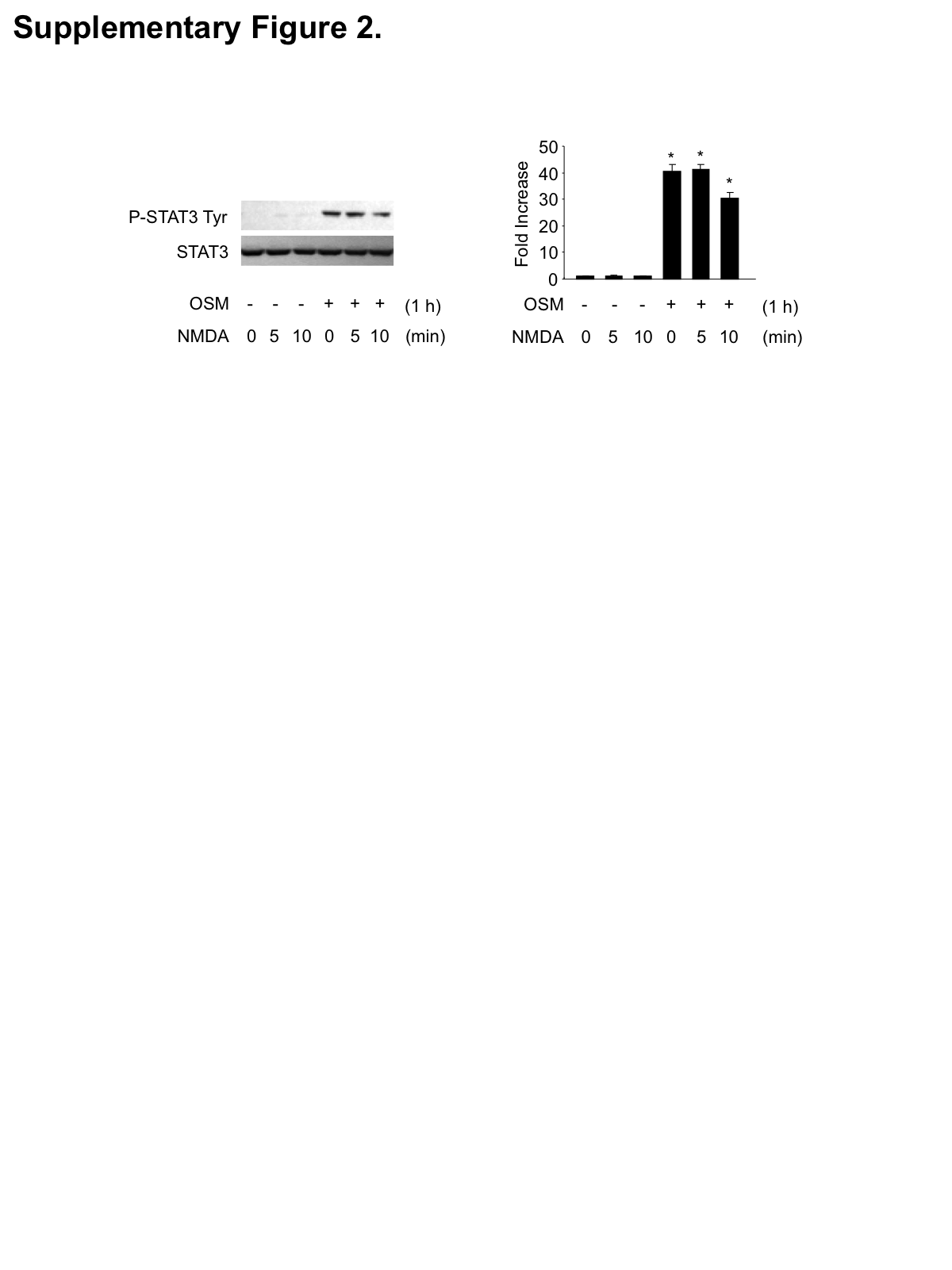

Supplement: Figure S2 — NMDA Does Not Affect STAT3 Activation in SH-SY5Y Cells. SH-SY5Y cells were pretreated for 1 h with OSM, followed by co-exposure of cytokine with NMDA (1 mM) for 5 and 10 min, and protein levels of phosphorylated STAT3 Tyr705 and total STAT3 were analyzed. The densitometric ratios of P-STAT3 Tyr705 versus total STAT3 were calculated, and shown as Fold Increase. Graph represents the mean ± SEM of triplicate cultures in three separate experiments. *p<0.001 compared to untreated cultures. (TIFF) [file pone.0050874.s002.tif]
